# Sub-microscopic magnetite and metallic iron particles formed by eutectic reaction in Chang’E-5 lunar soil

**DOI:** 10.1038/s41467-022-35009-7

**Published:** 2022-11-23

**Authors:** Zhuang Guo, Chen Li, Yang Li, Yuanyun Wen, Yanxue Wu, Bojun Jia, Kairui Tai, Xiaojia Zeng, Xiongyao Li, Jianzhong Liu, Ziyuan Ouyang

**Affiliations:** 1 Center for Lunar and Planetary Sciences, Institute of Geochemistry, Chinese Academy of Sciences, 550081 Guiyang, China; 2 Institute of Remote Sensing and Geographical Information System, School of Earth and Space Sciences, Peking University, 100871 Beijing, China; 3 College of Earth and Planetary Sciences, University of Chinese Academy of Sciences, 100049 Beijing, China; 4 Faculty of Metallurgical and Energy Engineering, Kunming University of Science and Technology, 650093 Kunming, China; 5 Center for Excellence in Comparative Planetology, Chinese Academy of Sciences, 230026 Hefei, China; 6 Guangdong University of Technology, 510006 Guangzhou, China; 7 State Key Laboratory of Continental Dynamics and Department of Geology, Northwest University, 710069 Xi’an, China

**Keywords:** Mineralogy, Geochemistry

## Abstract

Ferric iron as well as magnetite are rarely found in lunar samples, and their distribution and formation mechanisms on the Moon have not been well studied. Here, we discover sub-microscopic magnetite particles in Chang’E-5 lunar soil. Magnetite and pure metallic iron particles are embedded in oxygen-dissolved iron-sulfide grains from the Chang’E-5 samples. This mineral assemblage indicates a FeO eutectoid reaction (4FeO = Fe_3_O_4_ + Fe) for formation of magnetite. The iron-sulfide grains’ morphology features and the oxygen’s distribution suggest that a gas–melt phase reaction occurred during large-impact events. This could provide an effective method to form ubiquitous sub-microscopic magnetite in fine lunar soils and be a contributor to the presentation of ferric iron on the surface of the Moon. Additionally, the formation of sub-microscopic magnetite and metallic iron by eutectoid reaction may provide an alternative way for the formation of magnetic anomalies observed on the Moon.

## Abstract (web summary)

Magnetite is rarely present on the Moon. Here the authors report the magnetite formed by eutectic reaction during the impact process in Chang’E-5 lunar soil, and the potential contribution of this magnetite formation to magnetic anomalies on the Moon.

## Introduction

Traditionally, the Moon is considered to be extremely reduced. Thus, the oxidation state of the lunar surface points to formation of metallic iron rather than iron oxides^1^. Although recent remote sensing and sample analysis data indicate the presence of Fe^3+^ on the lunar surface, its distribution form and formation mechanism are unknown^2–4^. Magnetite is an important host mineral of Fe^3+^, and it is rarely present in lunar samples. In the Apollo era, there are some studies that deduced the presence of ubiquitous sub-microscopic magnetite-like phases in Apollo soils based on electron spin resonance and Mössbauer spectroscopy, but there is no further in situ mineralogical evidence for the presence of widespread magnetite crystals in lunar soils^5–8^. Some micron-sized magnetite grains have been identified in lunar samples, and they are closely associated with exogenous carbonaceous chondrite impactors, but these are isolated cases and there is no evidence for widespread distribution of magnetite grains in the finest lunar soils^2,9,10^. Therefore, the distribution forms of magnetite on the lunar surface remains a mystery, and the native magnetite products on the Moon are not known.

Lunar magnetic anomalies have been a mystery since the Apollo era^11–14^. Magnetite, an important ferromagnetic mineral, has not been considered as a carrier of lunar magnetic anomalies owing to the highly reducing conditions on the Moon^15,16^. Therefore, an in-depth understanding of the formation mechanism and distribution characteristics of magnetite on the Moon could provide a new perspective to explain the genesis of magnetic anomalies in the lunar crust.

China’s Chang’E-5 mission successfully returned 1.731 kg of new lunar soils from the young lunar mare basalt unit Em4/P58 (~2.0 Ga). Almost all of the Chang’E-5 regolith was local materials, with only a few distant ejecta (<5%) from large-impact craters (Aristarchus, Sharp B, Copernicus, and Harding)^17,18^. Considering that the Chang’E-5 ejecta formed at a young age and was subjected to very limited late modification processes, information about the initial response to large impact processes on the lunar surface can be obtained^19^. The discovery of high-pressure minerals in Chang’E-5 soils demonstrates the contribution of large-impact ejecta from the Chang’E-5 sampling region^20^.

Here, we report high-quality mineralogical analyses of native magnetite formed by eutectic reaction in Chang’E-5 samples. The newly formed sub-microscopic magnetite and metallic iron under large-impact conditions could greatly increase the magnetic susceptibility of lunar surface materials, which is a potential agent for giant-impact ejecta to exhibit significant magnetic anomalies.

## Results

### Overview of the iron-sulfide grains in Chang’E-5 lunar soil

**Fig. 1:**
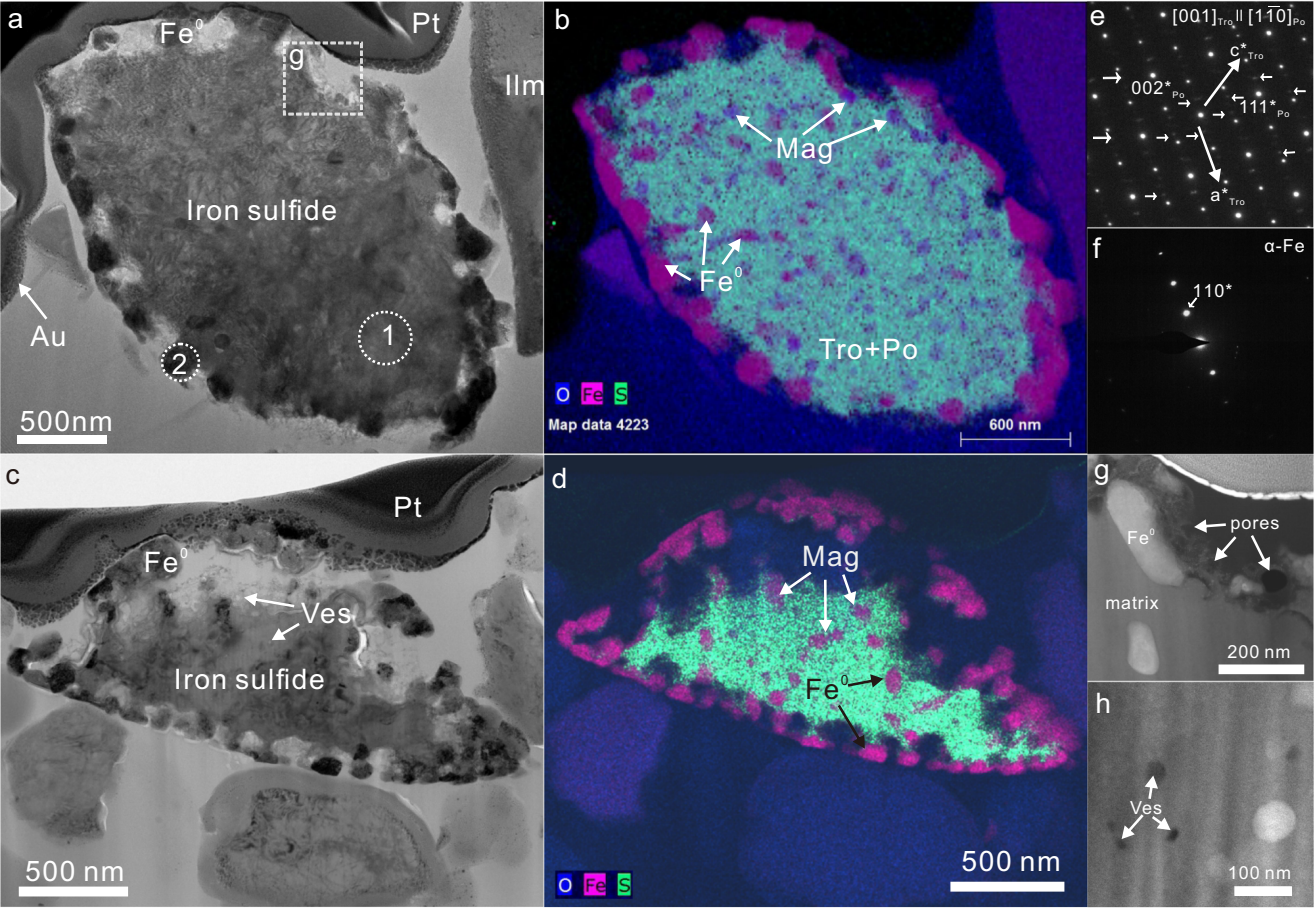
Overview of the studied spherical iron-sulfide grains. **a**–**d** Transmission electron microscopy bright-field images of two spherical iron-sulfide grains and their corresponding quantitative energy-dispersive element maps, showing a ring of equally spaced pure-iron tentacles at the grain edges and a wide distribution of iron-oxide (Mag) and pure-iron (Fe^0^) inclusions within the troilite (Tro) and pyrrhotite (Po) matrix. **e**–**f** Selected-area electron diffraction patterns of the matrix within an iron-sulfide grain and the iron tentacles around the grain edge, showing intergrowth of troilite and pyrrhotite in the matrix and metallic iron (α-Fe) protruding from the iron-sulfide grain. The diffraction area is indicated by the dashed region in (**a**). **g** Morphological features at the edges of the studied iron-sulfide grain. **h** Smaller vesicles (Ves) scattered within the grain.

X-ray diffraction and Raman spectroscopy results have shown that iron-sulfide is a minor component of Chang’E-5 soil samples^21–23^. Several angular and spherical iron-sulfide grains were selected by scanning electron microscopy (SEM) in our study (Supplementary Fig. 1). Angular troilite always exhibits sporadic curved iron whiskers with micro-scale length and a honeycomb-like mineral surface, as described by Matsumoto et al. (Supplementary Fig. 1)^24,25^. The angular troilite indicates that the grains were relatively homogeneous internally and did not exhibit complex mineral assemblages, suggesting that the grains were directly broken off from the parent rock without undergoing complex processes. In contrast, the spherical iron-sulfide grains (<2 μm in diameter), resembling molten droplets, showed unique morphological features in which the iron-rich components uniformly protruded from the entire surface of the iron-sulfide grains, showing a vermicular-like structure in the SEM images (Supplementary Fig. 1). Cross sections of the spherical iron-sulfide grains showed multiple mineral phases within the grains, which may provide additional evidence for the processes that occurred on the Moon (Fig. 1). We mainly focused on the spherical iron-sulfide grains, and the detailed information about the focused-ion-beam foils of these grains is given below.

**Fig. 2:**
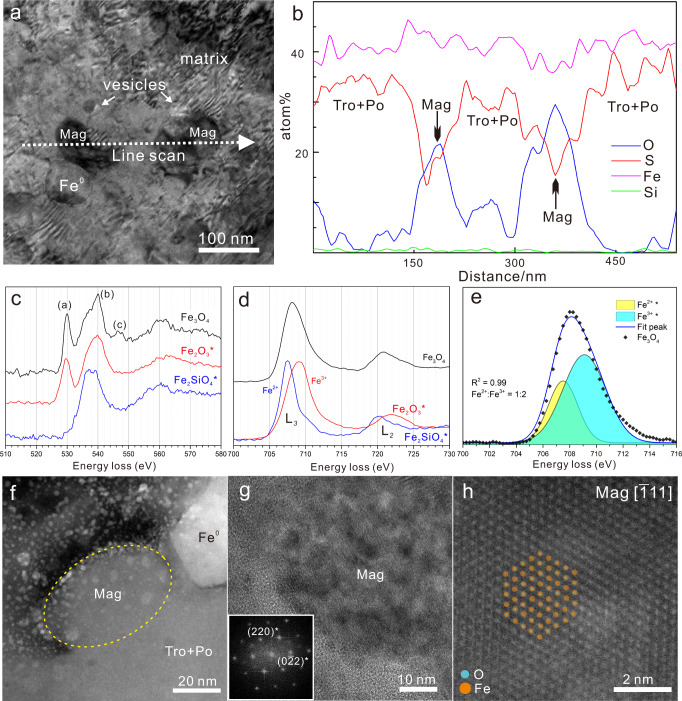
Identification of magnetite (Mag) in the iron-sulfide grains. **a** Transmission electron microscopy (TEM) bright-field image of a region of a spherical iron-sulfide grain focused-ion-beam section in which magnetite and pure metallic iron particles have co-precipitated within the troilite–pyrrhotite (Tro + Po) matrix with some scattered hexagonal vesicles. **b** Quantitative transmission electron microscopy–energy-dispersive line-profile results obtained from the position indicated by the arrow in (**a**). **c**, **d** O, K, and Fe *L*_2,3_ electron energy-loss spectra of a magnetite particle (Fe_3_O_4_) embedded in the studied iron-sulfide grain. The spectra of the prepared standards (Fe_2_O_3_ and Fe_2_SiO_4_) are marked by asterisks. **e** Results of the best fit (coefficient of determination of 0.99) for the Fe-oxidation-state ratio of the magnetite particle using the Fe *L*_3_ edge, showing an approximate Fe^3+^ to Fe^2+^ ratio of 2:1 in the magnetite particle. **f** High-magnification high-angle annular dark-field image of the magnetite particle, showing a close spatial relationship with metallic iron. **g** High-resolution TEM image of a magnetite particle. The fast Fourier transform pattern of this grain is shown in the insert at the bottom left of the figure. **h** Atomic-resolution annular dark-field scanning TEM image of a magnetite particle. A magnetite structure model along the [$\bar{{{{{{\bf{1}}}}}}}$11] zone axis is superimposed on the image.

Transmission electron microscopy (TEM) observations showed that the cross sections of the spherical iron-sulfide grains were elliptical with long and short axes of 2.5 and 1.5 μm, respectively, which is similar to the morphology of molten droplets (Fig. 1). Quantitative TEM-energy dispersive X-ray (EDX) compositional maps indicated that tentacles of pure iron (without Ni) protruded from the entire surface of the spherical iron-sulfide grains at nearly equal intervals (Fig. 1, Supplementary Fig. 2). In addition, the interiors of the grains contained abundant minute inclusions of pure metallic iron (undetectable Ni) and magnetite with sizes of ~100 nm, and the close spatial association of these two embedded phases suggested a co-precipitation formation (Figs. 1 and 2a). Based on selected-area electron diffraction (SAED) patterns and high-resolution TEM images of the iron-sulfide grains, the pure iron at the periphery of the particles and the interior iron particles were identified to be α-iron (Fig. 1, Supplementary Fig. 3). The matrix within the spherical iron-sulfide grains was identified to be intergrowth of troilite and pyrrhotite, with pyrrhotite exhibiting weak reflections in the SAED pattern. Troilite and pyrrhotite have a consistent topotaxial relationship, showing a [001] zone-axis of troilite parallel to pyrrhotite [1$\bar{1}$0] (Fig. 1e). The electron energy-loss spectroscopy (EELS) spectra and quantitative TEM-EDX results indicated that the iron-sulfide matrix also contained a certain amount of oxygen (Supplementary Fig. 4, Supplementary Table 1). TEM-EDX analysis gave an atomic Fe/S ratio of about 1.25 for the bulk composition of the interiors of the spherical iron-sulfide grains (including the magnetite, pure iron particles, and troilite–pyrrhotite matrix), indicating that there should be at least ~20% of excess elemental Fe within the iron-sulfide grains (Fe_1−*X*_S, *X* = 0–0.125) (Supplementary Table 1). Another feature of the spherical iron-sulfide grains was that the pure-iron tentacles at the grain edges were intertwined with strands of S- and O-rich material and contained numerous pores, with some areas containing Si and Ca (Supplementary Fig. 2). The pores at the edges of the grains were ~60 nm in diameter, suggesting that a violent outgassing reaction occurred at the edges of the grains (Fig. 1g). There were also some scattered smaller vesicles (~20 nm) within the grains (Fig. 1h).

### Identification of magnetite in the spherical iron-sulfide grains

The uniformly distributed iron-oxide particles with sizes of ~100 nm embedded in the spherical iron-sulfide grains were discovered to be magnetite by combined chemical and structural analyses. TEM-EDX compositional maps and line profiles indicated that the iron-oxide minerals within the spherical iron-sulfide grains were O- and Fe-rich phases (Figs. 1 and 2b). The EELS oxygen *K* edge at ~530 eV and iron *L*_2,3_ edge at ~705 eV are the clearest diagnostic features for identifying Fe_3_O_4_ in EELS spectra^26^. The EELS spectrum of the iron-oxide particles within the spherical iron-sulfide grains showed a pre-peak near 530 eV, a weaker maximum at ~545–550 eV, and a dominant peak at 540 eV, whose position and shape were between the standard O *K* edges of the Fe_2_SiO_4_ and Fe_2_O_3_ spectra (Fig. 2c). These detailed EELS spectral structures near 530 eV indicate the presence of Fe_2_O_3_ in the iron-oxide particles, and they have similar characteristics to those of Fe_3_O_4_^26^. In addition, the Fe *L*_2,3_ spectrum of the studied iron-oxide particles was intermediate between the standard single-valence spectra, indicating that both Fe^2+^ and Fe^3+^ were present in the iron-oxide particles within the spherical iron-sulfide grains (Fig. 2d). The Fe^3+^ to Fe^2+^ ratio in the iron-oxide particles was estimated to be ~2:1 by the EELS spectral peak fitting method, which is consistent with the chemical composition of magnetite (Fig. 2e). The structure of magnetite was further characterized by aberration-corrected scanning TEM. Atomic-resolution annular dark-field scanning TEM images reflecting the atomic mass contrast of the materials were obtained, with O and Fe showing relatively bright contrast in the images (Fig. 2h). High-resolution TEM images of the iron-oxide particles along the [$\bar{1}$11] zone axis indicated (220) lattice fringes with a periodicity of 2.97 Å (Fig. 2g). The angle between (220) and (0$\bar{2}$2) in the fast Fourier transform pattern was measured to be 120° (insert in Fig. 2g), which is consistent with the crystal structure of magnetite. The iron-oxide particles embedded in the spherical iron-sulfide grains were conclusively determined to be magnetite crystals.

## Discussion

The magnetite-bearing spherical iron-sulfide grains in the Chang’E-5 lunar fines were very small isolated grains (<2 μm in diameter). The spherical iron-sulfide grains were characterized by a ring of almost equidistant pure-iron tentacles at the edges of the grains, and by the ubiquitous sub-microscopic magnetite and metallic iron particles that precipitated in the internal troilite–pyrrhotite matrix. These spherical iron-sulfide grains with unique characteristics are an effective carrier of very small magnetite particles in lunar soils and a form of Fe^3+^ distribution on the lunar surface, providing verification of previous speculations about the ubiquity of very small magnetite particles in lunar soils^5–8,27^.

It is generally accepted that magnetite formation on the Moon is most likely to be controlled by the C–O–H gas phase under the reducing conditions of the lunar surface, and it is the most common interpretation of lunar surface magnetite in previous studies^2,10^. Joy et al. (2015) discovered a magnetite–troilite assemblage in Apollo samples. They inferred that there was a low-temperature equilibrium with oxidizing agents (H_2_O or CO_2_) from exogenous fluid-bearing impactors (carbonaceous chondrite or comets). This low temperature (~500 °C) origin of magnetite under oxidizing hydrothermal conditions has been used to explain formation of most magnetite in extraterrestrial samples, and it has been extensively studied by terrestrial rock experiments^2,10,28–32^. However, the magnetite found in this study coexisted with metallic iron and iron-sulfide minerals. These phases correspond to quite different oxygen fugacity conditions, and thus C–H–O fluid alteration processes are not responsible for formation of magnetite in the studied spherical iron-sulfide grains^33^. Magnetite–FeNi–iron sulfide assemblages have been found in meteorites, which has been attributed to the high-pressure and high-temperature conditions that allow them to coexist^34,35^.

The main difference between our observations and the phenomena reported in the studies mentioned above is that it is pure metallic iron particles that coexisted with sub-microscopic magnetite in the Chang’E-5 samples. This indicates another formation mechanism of magnetite that is generally accepted in the field of hot-rolled steel sheets, namely, co-precipitation of magnetite and metallic iron from iron oxides by a eutectoid reaction^28,36–38^. The eutectoid reaction can be simply described as decomposition of metastable wüstite (FeO) to magnetite (oxidized species) and metallic iron (reduced species) at a relatively low temperature of ~570 °C^39–41^.

The excess elemental Fe and O in the studied iron-sulfide grains, as well as the volume of the vesicles was much less than the total volume of pure-iron and magnetite particles within the iron-sulfide grains, which suggest that a certain amount of FeO is dissolved within iron-sulfide (FeS + 6FeO = SO_2_ + 4Fe + Fe_3_O_4_). The ellipsoidal shape, large number of pores at the grain edges, and formation of pure metallic iron all indicate that the studied spherical iron-sulfide grains experienced high-temperature events.

Numerous observations and simulations of extraterrestrial sample have demonstrated that a considerable amount of non-siderophile O components from the surrounding O-containing matrix can be incorporated into the metal-sulfide phase and form Fe–S–O systems under high-temperature conditions during impact processes^35,42–44^. Leroux et al. (2000) reported that the metal-sulfide globules embedded in an amorphous silicate-glass matrix contained 13 wt% of FeO under melting conditions, and the phase diagram showed that the melting point of the Fe–S–O system decreases with addition of the O component^35,45^. Based on the oxygen content of the Chang’E-5 spherical iron-sulfide grains, thermodynamic calculations showed that the ~20% of dissolved FeO per mole of FeS in the iron-sulfide grain should experience a temperature of above 915 °C (Supplementary Fig. 5). Therefore, the studied Chang’E-5 spherical iron-sulfide grains underwent melting and formed ellipsoidal grains.

The FeO–FeS phase diagram was used to constrain magnetite and pure metallic iron particles formation within the spherical iron-sulfide grains, yielding a eutectic temperature of α-Fe, magnetite, and pyrrhotite of below 600 °C (Supplementary Fig. 6). The magnetite and pure metallic iron particles were commonly euhedral and entirely embedded in the studied spherical iron-sulfide grains, showing that they formed as solid-state precipitates at conditions below the melting point^46^. At the edges of the grains, there was no magnetite in equal proportions to the pure-iron tentacles, suggesting a different reaction process to that within the spherical iron-sulfide grains. Such massive production of pure metallic iron accompanied by a large number of pores can be interpreted as desulphurization of troilite (2FeS = 2Fe + S_2_) or reduction of iron oxide and iron-sulfide to metallic iron (2FeO + FeS = 3Fe + SO_2_)^2,47^. The lunar vacuum conditions allow this reaction to continue to outgas and lead to formation of large amounts of pure iron wrapped around the grain edges. The scattered vesicles within the grain can also be attributed to these reactions.

The absence of other molten phases attached to the spherical iron-sulfide grains and the filamentous amorphous Si–Ca–S–O composition with a vesicular structure detected at the edge of the grain suggest that the molten iron-sulfide droplets most likely interacted with the silicate gas. The occurrence of vapor phase in lunar surface conditions involved temperature far >2000 °C, which is indicative of large-impact processes on the Moon^47,48^. Furthermore, simultaneous precipitation of magnetite and metallic iron within the Chang’E-5 iron-sulfide grains indicates the conditions for equilibrium crystallization. Thus, the grains experienced an extremely high-temperature environment for a long duration, and such spherical iron-sulfide droplets are more likely to be produced in oxygen-bearing gas columns formed by large impacts^47^.

Based on the above discussion, a plausible scenario for the complex phenomenon within the spherical iron-sulfide grain is that the molten iron-sulfide droplets reacted with the surrounding silicate vapor during a large-impact event. During the melt stage of iron-sulfide, the surrounding FeO-gas component reacted with the edges of the iron-sulfide droplets in a reduction reaction, forming a substantial amount of iron tentacles around the grains, and some of the FeO gas dissolved in the interior of the iron-sulfide droplets. During the subsequent rapid-solidification process, the FeO dissolved inside the iron-sulfide droplet decomposed to form sub-microscopic magnetite and pure metallic iron particles.

The newly discovered spherical iron-sulfide grains in the Chang’E-5 lunar fines contained sub-microscopic metallic iron and magnetite particles, both of which are important ferromagnetic minerals with magnetic susceptibilities of 20,000–110,000 and 50,000, respectively, compared with 13–36 for troilite. Therefore, the eutectic reaction that occurred within the iron-sulfide minerals during impact may significantly improve the magnetic properties of the lunar crustal materials^16^.

Lunar magnetic anomalies have been a mystery since the Apollo era, and their origin is still under debate^12,13^. Orbiting magnetometer data from Lunar Prospector suggests that magnetic anomalies may be associated with impact basins on the Moon, especially because lunar surface impact ejecta deposits are often strongly magnetized^12,13,49^. Generation of magnetic anomalies on the lunar surface mainly depends on the content of ferromagnetic minerals in the lunar soil and the strength of the external magnetic field, and stronger magnetic properties can be recorded for greater degree of magnetization of the lunar surface minerals^15^. In addition to the existence of the lunar core dynamo field (~3.9 Ga), impact processes have been demonstrated to be a key pathway for magnetic field generation in the lunar crust^13,50–52^. However, formation and the distribution of ferromagnetic minerals that can effectively contribute to the magnetic anomalies on the lunar surface are still unclear, and it is difficult to link the magnetic properties of known endogenous lunar materials to lunar crustal anomalies because of the ~2–4 order of magnitude weaker magnetic properties relative to terrestrial materials^15^.

Wieczorek et al. (2012) performed numerical simulations of large-scale impacts. They found that chondritic projectile materials (Fe metal) from giant impacts can provide the highly ferromagnetic minerals to account for the intensity of the observed magnetic anomalies, and the simulations were consistent with the magnetic properties of distal ejecta from the South Pole–Aitken basin formation event^12^. Therefore, impact-related materials may be the most plausible carriers of magnetic anomalies. It is well known that the extremely high temperature and pressure conditions provided by large impacts are necessarily accompanied by significant material transformations^53,54^. However, in addition to ferromagnetic materials directly injected by the impactor (e.g., FeNi), newly formed ferromagnetic minerals during the large-impact have not been considered. The results of this study of Chang’E-5 lunar soil indicate that iron-sulfide minerals undergo complex eutectic reactions during impacts to form highly ferromagnetic minerals (sub-microscopic magnetite and metallic iron), which could also be an important source of ferromagnetic material on the lunar surface. Considering that iron-sulfide is an important component of chondritic meteorite projectiles, it is highly likely that this reaction occurred during large impacts on the lunar surface. Since the high magnetic susceptibilities of magnetite and metallic iron, the impact process would greatly reduce the thickness requirements of lunar soil for lunar magnetic anomalies, irrespective of whether the ferromagnetic minerals are brought directly from the impactor body or such reaction acts on iron-sulfide minerals^12^.

We mapped the total magnetic field strength at the lunar surface based on Kaguya and Lunar Prospector magnetometer data. The Chang’E-5 landing site exhibits a relatively low intensity with an estimated maximum magnetic field strength of 1.18 nT (Supplementary Fig. 7)^55,56^. Our observations showed that there were very few spherical iron-sulfide grains in the Chang’E-5 lunar fines, and only two magnetite-bearing spherical iron-sulfide grains were found in the studied samples. We suggest that the two main reasons for the weaker magnetic field strength in the Chang’E-5 region are as follows: (1) Lunar magnetic field anomalies are spatially correlated with large-impact ejecta, but only a few distant ejecta from large-impact craters are mixed in the Chang’E-5 sampling region^23^. (2) Chang’E-5 lunar soil has a young age of formation, and the possibility of the presence of an effective external magnetic field (generated by ancient core dynamics or basin-forming impacts) is relatively low.

Based on the above discussion, we conclude that the key factors for generation of magnetic anomalies on the lunar surface are (1) the presence of ferromagnetic minerals originating from large-impact events, including projectile injection or impact-induced material transformation, and (2) magnetization of the ferromagnetic minerals in the presence of external magnetic fields (impact-related magnetic fields or core dynamo fields). These formation conditions result in a matching relationship between the magnetic anomaly distribution in the lunar crust and the distal ejecta of large impacts.

## Methods

### Samples

The Cheng’E-5 samples examined in this study consisted of the fine fractions of lunar regolith soils (CE5C0400YJFM00505 and CE5C0200YJFM00302). The lunar soils were spread on silicon wafer and covered with a gold film for the SEM observations.

### SEM and transmission electron microscopy (TEM) analysis

The iron-sulfide grains were initially identified by backscatter electron imaging, which showed a bright contrast, and they were confirmed by EDX combined with field-emission SEM (FEI Scios) at the Institute of Geochemistry, Chinese Academy of Sciences (CAS), Guiyang (Supplementary Fig. 1). Observation of the nanophases within the focused-ion-beam samples was performed by field-emission scanning TEM (200 kV, FEI Talos F200X) at the Suzhou Institute of Nano-tech and Nano-bionics, CAS. Chemical analysis of the focused-ion-beam foils was performed by scanning TEM combined with energy-dispersive detection. High-resolution TEM images and SAED patterns were acquired to identify the nanocrystal structure.

### Electron energy-loss spectroscopy (EELS) analysis

EELS spectra were employed to measure the oxidation state of Fe in the nanocrystals. EELS analyses were performed using a Gatan GIF Quantum ER System Model 965 parallel EELS spectrometer attached to a Hitachi HF5000 aberration-corrected scanning transmission electron microscope housed at the Shanghai Institute of Ceramics, CAS, operating at an accelerating voltage of 200 kV. We collected EELS spectra in DualEELS mode with a probe current of 100 pA. The energy resolution was between 0.5 and 0.7 eV, as measured from the full width at half maximum height of the zero-loss peak. The line profiles of EELS were acquired at 0.25 eV/channel dispersion with a dwell time of 18 s/point for O and Fe, and the acquisition times were 10 s for the Fe and O point analyses. Fayalite and hematite standards were prepared as Fe^2+^ and Fe^3+^ references to quantitatively calculate the Fe^2+^/Fe^3+^ ratios of magnetite from the EELS spectra. The Fe-oxidation-state ratios were quantified using the *L*_3_ edge, and the peak positions and full widths at half maximum heights of the standards were used as constraints for the fit. The Fe-oxidation-state ratios of magnetite were determined by normalizing the best-fit weight, and the goodness of fit was evaluated using the coefficient of determination. The spectra of magnetite with coefficients of determination of ~0.99 (with a value closer to 1 indicating a better fit) are reported in our study.

### Lunar magnetic field strength mapping

The data of the total field intensity on the lunar surface were derived from the results of Tsunakawa et al. (2015) and Ravat et al. (2020)^55,56^. Originally, the magnetic field was obtained at altitudes of 10–45 km by the Kaguya and Lunar Prospector missions. The surface components were derived by surface-vector mapping in Tsunakawa et al. (2015) and by L1-norm model regularization of the radial component at the surface on the magnetic monopole bases and along-track magnetic field differences in Ravat et al. (2020)^55,56^.

## Supplementary information

Supplementary Information

Peer Review File

## Data Availability

All data are available in the main text or the supplementary information, and the original TEM and EELS data of this study are available in Guo (2022)^57^ and online at https://data.mendeley.com/datasets/nd2tc5bykb/1.
