# Supplementary material for: Sub-microscopic magnetite and metallic iron particles formed by eutectic reaction in Chang’E-5 lunar soil
Source: Nat Commun. 2022 Nov 23;13:7177. doi: 10.1038/s41467-022-35009-7 (PMC9684415; doi:10.1038/s41467-022-35009-7)
Supplement: Supplementary file 1 — Supplementary Information [file 41467_2022_35009_MOESM1_ESM.pdf]

Supplementary information for

Sub-microscopic magnetite and metallic iron particles formed by eutectic reaction in Chang'E-5 lunar soil

Zhuang Guo[1,2,3], Chen Li[1,4], Yang Li[1,5]*, Yuanyun Wen[1], Yanxue Wu[6], Bojun jia[2], Kairui Tai[1,7], Xiaojia Zeng[1], Xiongyao Li[1,5], Jianzhong Liu[1,5], Ziyuan Ouyang[1]

[1] Center for Lunar and Planetary Sciences, Institute of Geochemistry, Chinese Academy of Sciences, Guiyang 550081, China

[2] Institute of Remote Sensing and Geographical Information System, School of Earth and Space Sciences, Peking University, Beijing 100871, China

[3] College of Earth and Planetary Sciences, University of Chinese Academy of Sciences, Beijing 100049, China

[4] Faculty of Metallurgical and Energy Engineering, Kunming University of Science and Technology, Kunming, 650093, China

[5] Center for Excellence in Comparative Planetology, Chinese Academy of Sciences, Hefei 230026, China

[6] Guangdong University of Technology, Guangzhou 510006, China

[7] State Key Laboratory of Continental Dynamics and Department of Geology, Northwest University, Xi'an 710069, China

*Corresponding Author. E-mail address: liyang@mail.gyig.ac.cn

This file includes:

Supplementary Figs. 1-7
Supplementary Table 1

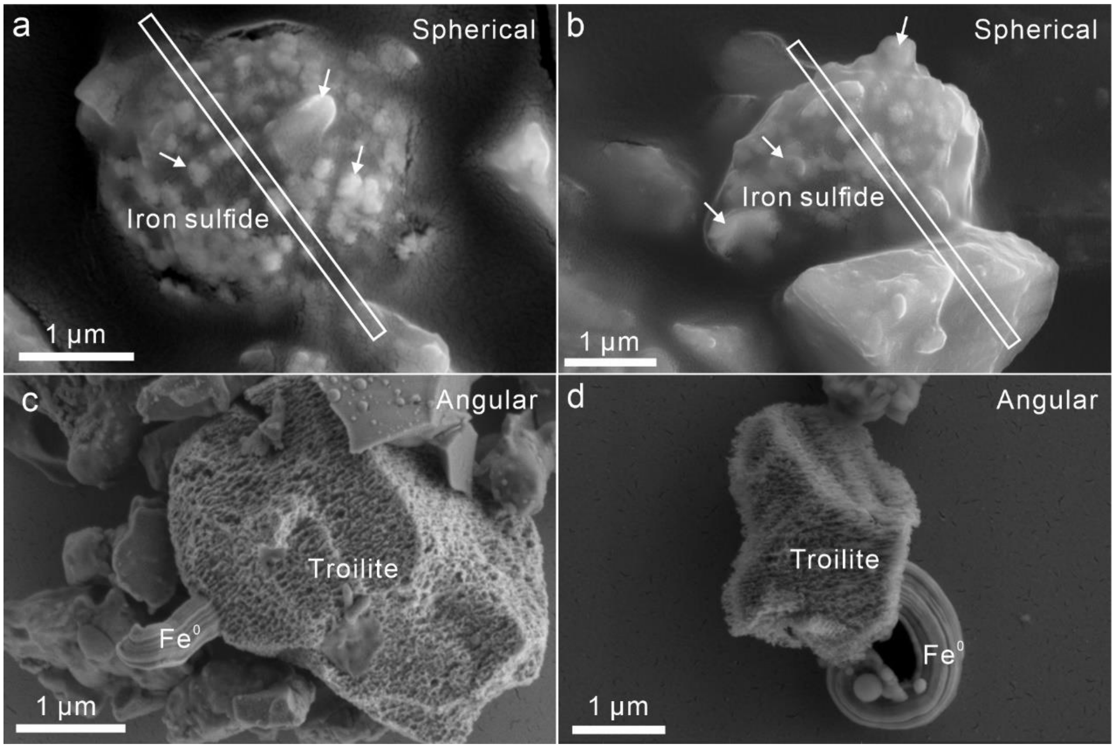

Supplementary Fig. 1. The morphology of iron-sulfide grains in Chang'E-5 lunar soil. (a-b) Scanning electron microscope (SEM) images of spherical iron-sulfide grains from the fine fraction of the Chang'e-5 lunar soils (c-d) SEM images of angular iron-sulfide grains in Chang'E-5 lunar soil. The white rectangles indicate the extraction locations of the focused ion beam sections.

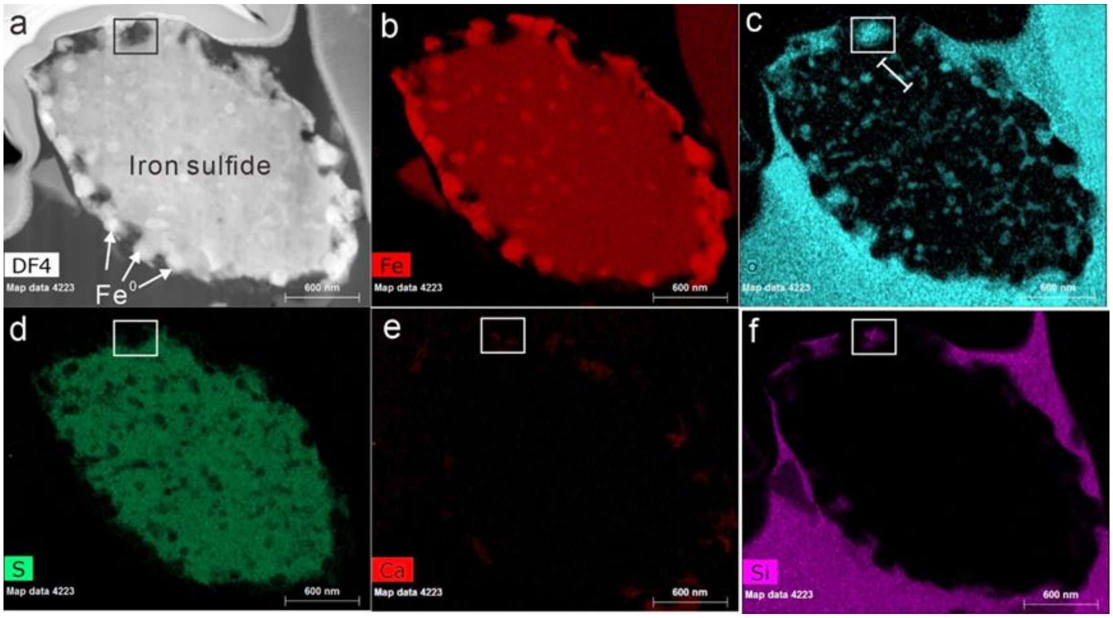

Supplementary Fig. 2. TEM-energy dispersive X-ray (EDX) compositional maps of the spherical iron-sulfide grain. (a-f) High-angle annular dark-field image (a) of spherical-like iron sulfide and corresponding quantitative TEM-EDX Fe (b), O (c), S (d), Ca (e), and Si (f) element maps. Rectangular regions reveal the composition of interstitial material between the pure iron tentacles. The line in Supplementary Fig. 2c represents the location of the EELS line-scan of Supplementary Fig. 4.

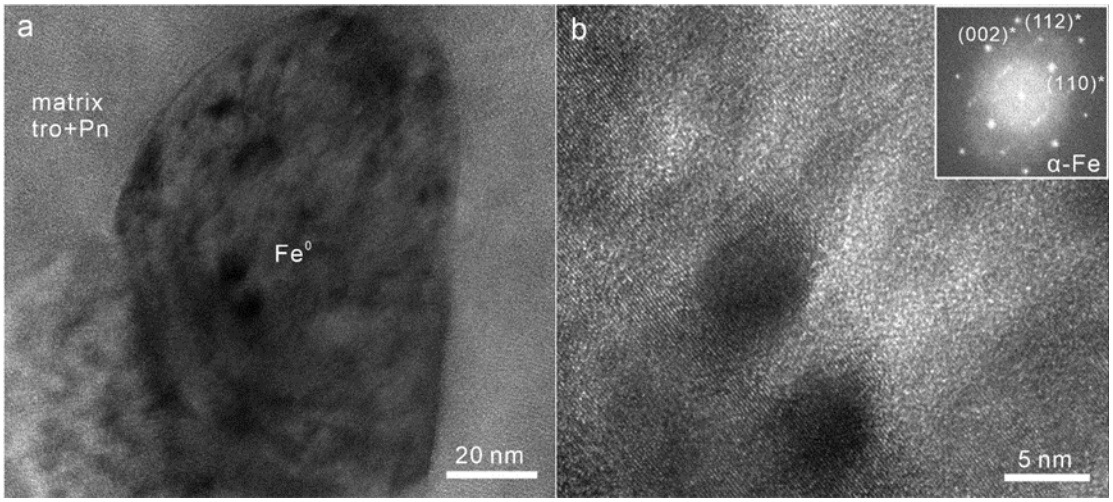

Supplementary Fig. 3. The structure of pure metallic iron within the iron-sulfide grains. (a) A close-up view under TEM bright-field imagery. (b) High resolution (HR) TEM image of the pure metallic iron within the iron sulfide grains, showing the α-Fe structure.

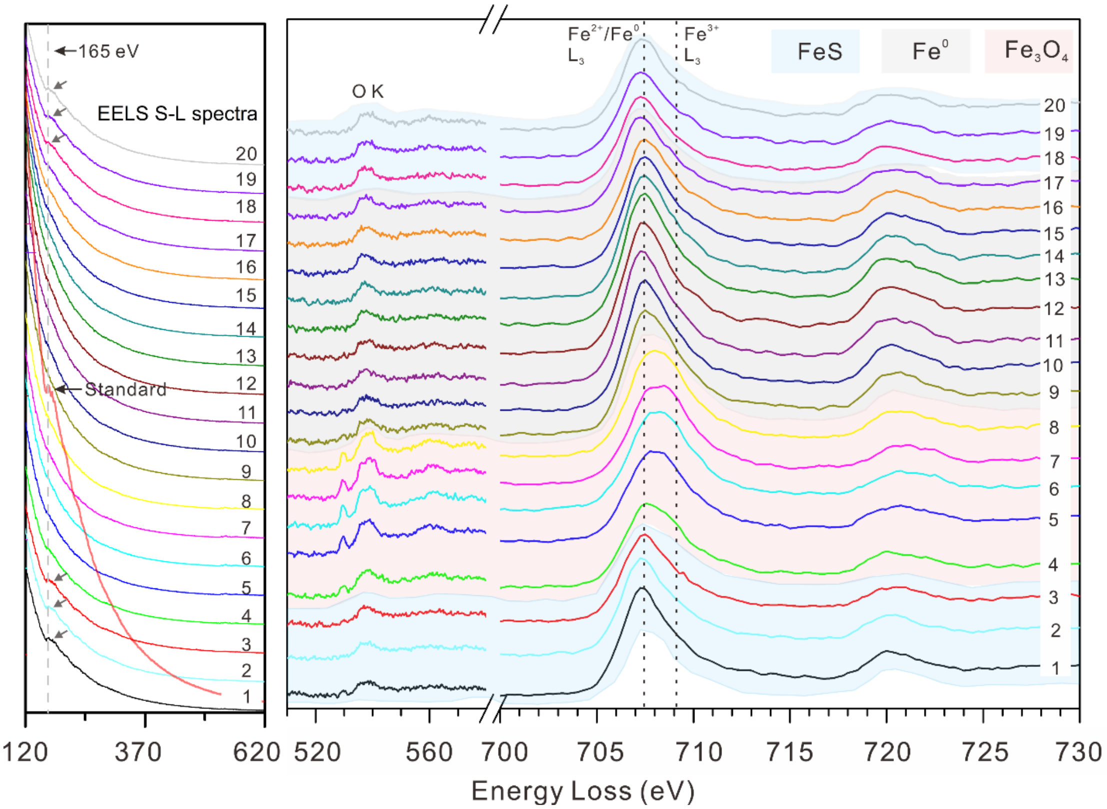

Supplementary Fig. 4. EELS spectrum of S, Fe, and O from the line-scan (Fig. S3c). The line passes through the mineral phases of matrix, pure metallic iron, and the magnetite within the iron sulfide.

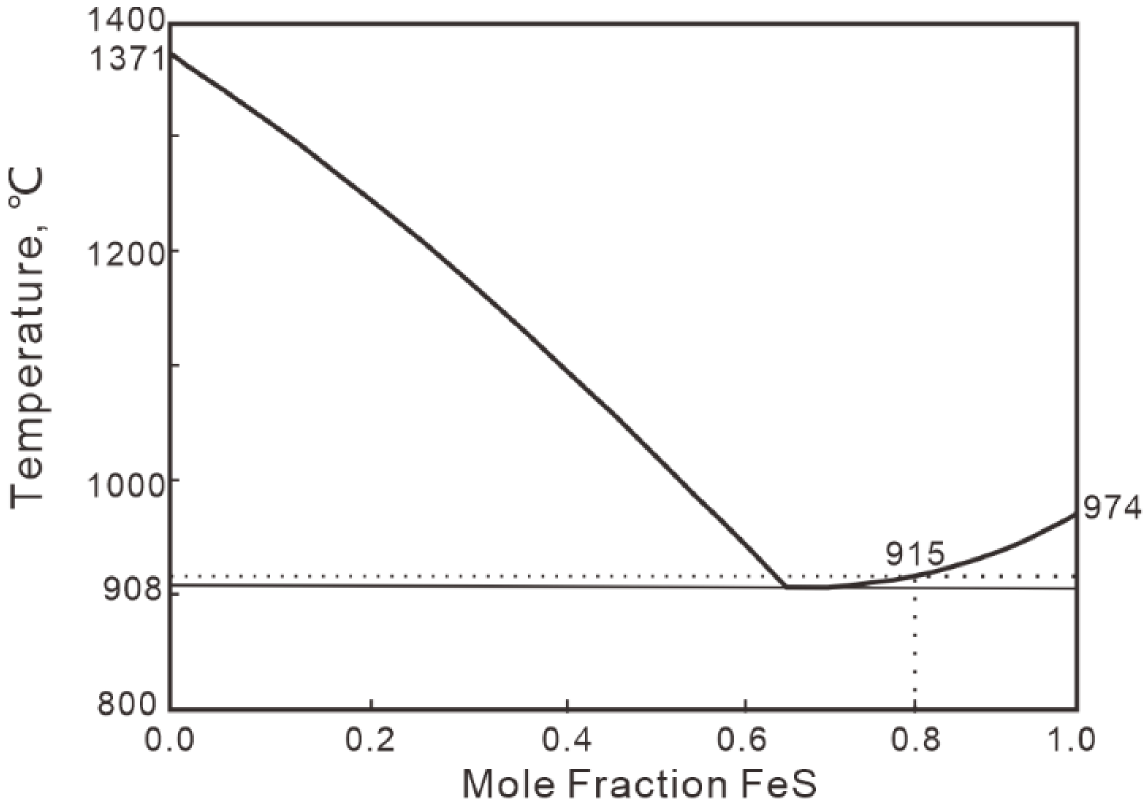

Supplementary Fig. 5. The Fe-S-O phase diagram. The diagram indicates a melting point of about 915 ℃ for a mole fraction FeS of approximately 80 %.

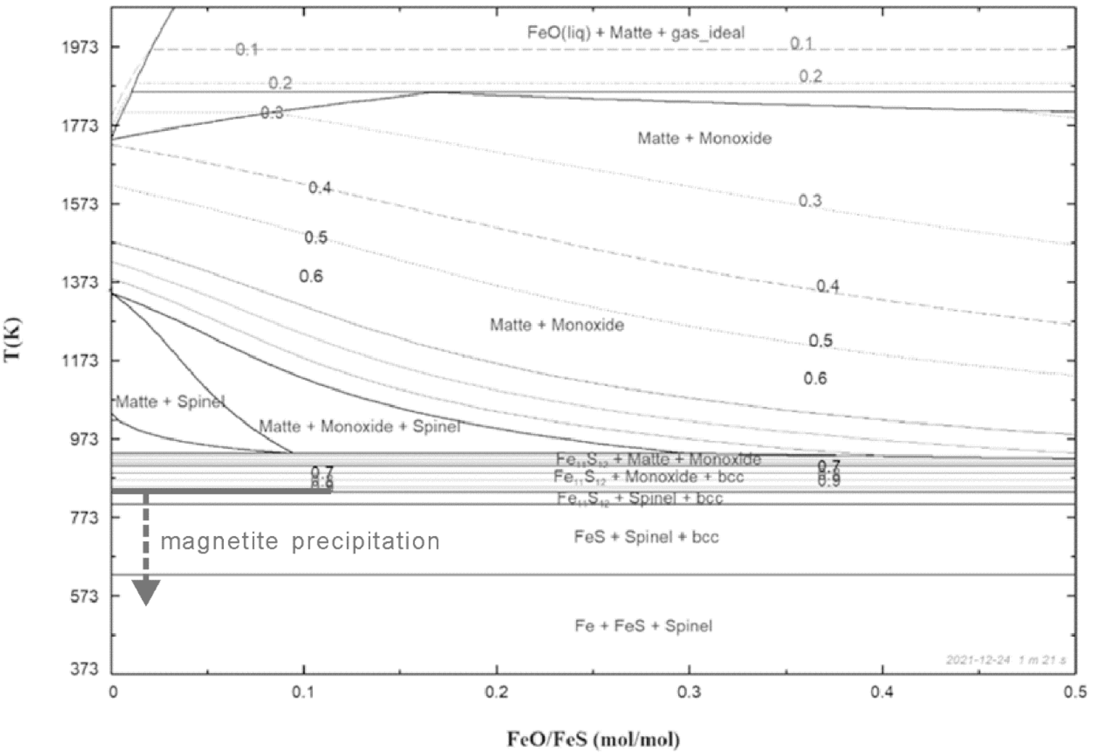

Supplementary Fig. 6. The phase diagram of FeO-FeS system. This phase diagram indicates that the eutectic temperature of α-Fe, magnetite, and pyrrhotite should be below 600℃.

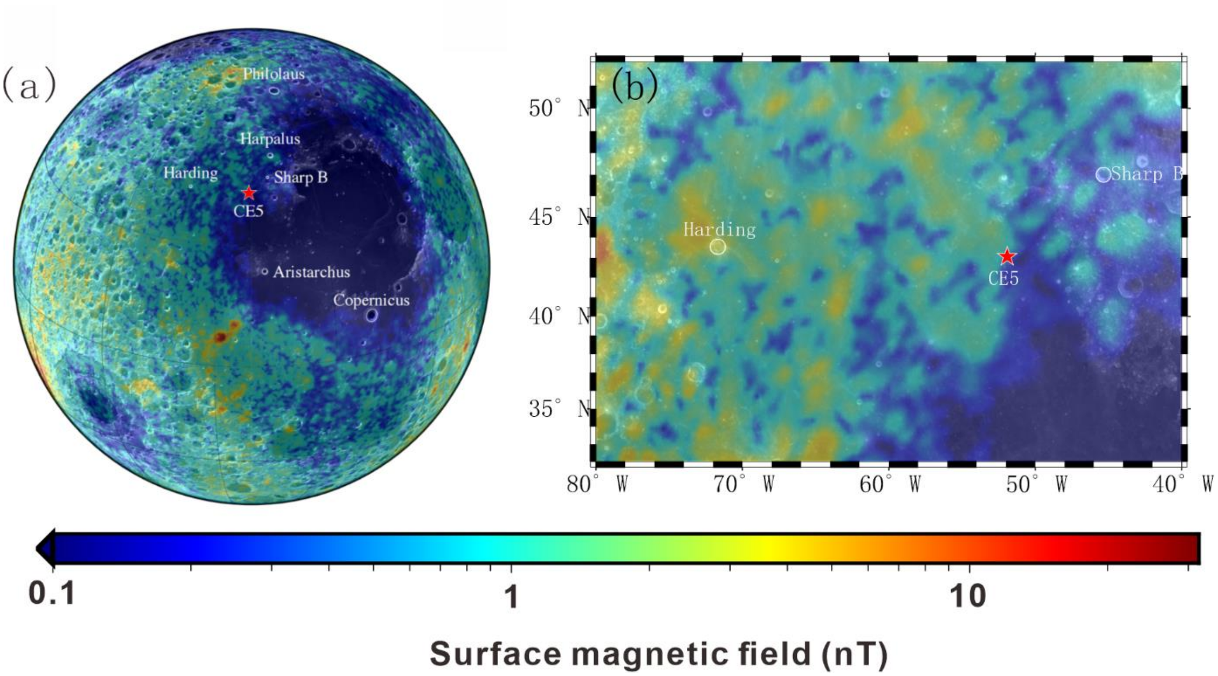

Supplementary Fig. 7. Lunar magnetic field strength mapping. (a) Magnetic field strength at the lunar surface based on observations from Kaguya and Lunar Prospector magnetometers. (b) Magnetic field strength of the Chang'e-5 landing region, which exhibits a relatively low magnetic field strength.

Supplementary Table 1. Quantitative TEM EDS analyses of various phases in the interior of spherical iron sulfides.

	Bulk (6)	Fe^0 (4)	Matrix (Tro + Pn) (10)	Magnetite (5)
O	4.30	0.36	1.57	29.9
Fe	42.1	78.6	41.52	38.4
S	33.2	0.53	36.34	13.9
Si	0.46	0.64	0.43	0.50
Al	0.73	1.12	0.75	0.49
Ca	0.24	0.23	0.32	0.26
Mg	0.52	0.23	0.52	0.53
Ti	0.22	0.23	0.21	0.20
Ni	0.03	0.05	0.04	bd
S/Fe	0.79	-	0.88	-

Note. bd = below detection limits;

Number in brackets is the number of EDS analyses performed.

All data is the initial measured data (at%).
